# Comparison of photon intensity modulated, hybrid and volumetric modulated arc radiation treatment techniques in locally advanced non-small cell lung cancer

**DOI:** 10.1016/j.phro.2023.100519

**Published:** 2023-11-18

**Authors:** Djoya Hattu, Daisy Emans, Judith van der Stoep, Richard Canters, Judith van Loon, Dirk De Ruysscher

**Affiliations:** Department of Radiation Oncology (Maastro), GROW School for Oncology and Reproduction, Maastricht University Medical Centre, Maastricht, The Netherlands

**Keywords:** Non-small cell lung cancer, Treatment techniques, IMRT, VMAT, NTCP models, Cardiac substructures

## Abstract

**Background and purpose:**

There is no consensus on the best photon radiation technique for non-small cell lung cancer (NSCLC). This study quantified the differences between commonly used treatment techniques in NSCLC to find the optimal technique.

**Materials and methods:**

Treatment plans were retrospectively generated according to clinical guidelines for 26 stage III NSCLC patients using intensity modulated radiation therapy (IMRT), hybrid, and volumetric modulated arc therapy (VMAT_C_, and VMAT_V5_ optimized for lower lung and heart dose). Plans were evaluated for target coverage, organs at risk dose (including heart substructures) and normal tissue complication probabilities (NTCP).

**Results:**

The comparison showed significant and largest median differences (>1 Gy or >5%) in favor of IMRT for the mediastinal envelope and heart (maximum dose), in favor of the hybrid technique for the lungs (V5Gy of the total lungs and V5Gy of the contralateral lung) and in favor of VMAT_C_ for the heart (Dmean), most of the substructures of the heart, and the spinal cord (maximum dose). The VMAT_V5_ technique had significantly lower heart dose compared to the hybrid technique and significantly lower lung dose compared to the VMAT_C_, combining both advantages in one technique. The mean ΔNTCP did not exceed the 2 percent point (pp) for grade 5 (mortality), and 10 pp for grade ≥2 toxicities (radiation pneumonitis and acute esophageal toxicity), but ΔNTCP was mostly in favor of VMAT_C/V5_ for individual patients.

**Conclusion:**

This planning study showed that VMAT_V5_ was preferred as it achieved low lung and heart doses, as well as low NTCPs, simultaneously.

## Highlights

• Dose was evaluated for all important organs at risk, including the substructures of the heart.
• Dose to the substructures of the heart was lowest with the Volumetric Modulated Arc Techniques.
• The Volumetric Modulated Arc Technique was able to achieve low lung and heart doses simultaneously.
• Median differences in Normal Tissue Complication Probability between the techniques was less than 2 pp for grade 5 toxicity and 10 pp for grade ≥2 toxicity.

## 1 Introduction

Radiotherapy combined with chemotherapy, followed by durvalumab immunotherapy, is the standard of care for fit inoperable stage III non-small cell lung cancer (NSCLC) patients [1]. In the PACIFIC study, on which this new standard of care is based [2], different types of photon therapy techniques were used, including 3D conformal radiotherapy (3DCRT), intensity-modulated radiation therapy (IMRT), volumetric-modulated arc therapy (VMAT) and hybrid configurations [3], [4].

Although intensity modulated techniques allow for delivery of high-dose radiotherapy, even in patients with bulky tumors [5], there is inconclusive data to estimate the difference between the different techniques for reduction of toxicities in lung cancer [6], [7], [8], [9], [10], [11], [12], [13], [14], [15], [16], [17]. Radiation induced toxicities in lung cancer patients consist mainly of radiation pneumonitis (RP), acute esophageal toxicity (AET) and cardiac toxicity, related to the dose to the lungs, the esophagus and parts of the heart [18], [19], [20], [21], respectively, as well as the functional status of the heart [22]. Dose constraints for these organs at risk (OARs) are considered as objectives during inverse treatment planning and compete with each other in the optimization function. It remains challenging to determine which dose differences are clinically relevant. Normal tissue complication probability (NTCP) models are used to predict the probability of these side effects and can assist in the evaluation of a treatment plan [23], [24], [25].

There is renewed emphasis on lowering heart dose to reduce cardiac toxicities [26], [27]. However, few studies include all relevant OARs and/or substructures of the heart [28]. Some studies incorporate NTCP models in the treatment technique planning comparison, but for other treatment sites [29], [30], [31]. It is important to evaluate the current treatment planning techniques by including these new insights. The aim of this study was to perform a comprehensive comparison of contemporary treatment techniques for NSCLC patients combining all these aspects: evaluating dose differences for target coverage and dose to all relevant OARs, including heart substructures, and estimating the clinical relevance of the observed dose differences by incorporating clinically implemented lung cancer NTCP models.

## 2 Materials and methods

This study included 26 stage III NSCLC patients, covering different tumor locations in the lung (see Table 1 for patient characteristics).

**Table 1:** Patient characteristics. GTV: Gross Tumor Volume, PTV: Planning Target Volume, IQR: Interquartile range.

			**Counts (n)**	**%**
	Clinical Stage	IIIa	8	31
		IIIb	18	69
	Clinical Tumor stage	1, 1a, 1b	1	04
		2, 2a, 2b	7	27
		3	7	27
		4	11	42
	Clinical Nodal stage	2	26	100
	Tumor location	Left upper lobe	7	27
		Left lower lobe	5	19
		Right upper lobe	7	27
		Right middle lobe	1	4
		Right lower lobe	6	23
	Lymph node stations	2, 3, 4 (superior mediastinal)	30	115
		5, 6 (aortic)	6	23
		7, 8, 9 (inferior mediastinal)	14	54
		10, 11 (pulmonary)	17	65

			**Volume (cc)**	**IQR**

	GTV volume	Median	88.6	43–113
	PTV volume	Median	415.9	269–494

### 2.1 Imaging and structure delineation

Patients were positioned on a chest board (Macromedics) with their arms above their head and a knee support (Civco). A 4DCT (Siemens SOMATOM Confidence RT Pro, 3 mm slice thickness) was acquired, as well as a PET and contrast CT to guide the target volume delineation. Gross tumor volumes for the primary tumor (GTVp), lymph nodes (GTVn) and the OARs (lungs, heart, esophagus, mediastinal envelope, spinal cord) were delineated on the 50% expiration phase of the 4DCT. Primary and nodal CTVs were defined by a 5 mm isotropic expansion of their respective GTVs, and adapted to the surrounding normal tissue. The planning target volume for the primary tumor (PTVp) was at least 8 mm, and dependent on the tumor amplitude to account for motion. PTVn was a 5 mm isotropic margin of CTVn. The lungs were defined as the total lungs minus the total GTV. The esophagus was delineated from the end of the larynx to the gastroesophageal junction, and included the outer muscle layer. The heart was delineated according to the RTOG guidelines starting from the apex and included all major vessels [32]. The mediastinal envelope enclosed the heart, the esophagus, all arteries and veins, proximal bronchi and trachea at the level of the PTV. The mediastinum was expanded with a 5 mm margin to create a planning risk volume (PRV) [33].

### 2.2 Treatment planning techniques

All 26 patients were planned with three photon treatment techniques: 1. a sliding window IMRT technique, 2. a hybrid technique and 3. two VMAT techniques. The IMRT plans consisted of five fields with a multileaf collimator (MLC) sliding window technique. Treatment fields were positioned at the affected side of the patient. Hybrid plans consisted of two static fields (one from anterior and one from posterior) complemented with at least one but predominantly two 180 degree VMAT beams, depending on the size of the target volume. The static fields covered a maximum of 2/3 of the total dose. The two VMAT techniques consisted of a clinically used VMAT technique (VMAT_C_) and a VMAT technique with stricter objectives for the V5Gy of the total lungs and dose to the contralateral lung (VMAT_V5_). The latter technique was developed to investigate if lower lung doses were possible with a full VMAT technique while maintaining low heart doses. Supplementary Fig. 1 shows the difference in objective values between the two VMAT plans. Both VMAT plans consisted of two 6MV fields with beam angles of 0–179 (left sided) or 0–181 (right sided). To ensure a fair comparison, treatment plans were generated in a research version of Eclipse (Acuros dose engine v15.5.11, 2.5 mm dosegrid, TrueBeam STx machine, Varian Medical Systems, Palo Alto) and randomly assigned to experienced RTTs who followed a study planning protocol that included beam setup and dose requirements for target coverage and OARs (Supplementary Table 1). The optimization objective priorities were the same for all treatment techniques, with the exception of the VMAT_V5_ plans that aimed for a further reduction of the lung dose. The prescribed dose to the PTV was 60 Gy in 30 fractions and treatment plans were normalized to ensure that 95% of the PTVn and PTVp volume was covered by 95% of the prescribed dose.

### 2.3 DVH analysis

Evaluation of the PTV concerned a comparison of the D2% (maximum dose), D98% (minimum dose), HI (homogeneity index) and CN (conformation number). The HI was calculated using the formula $\mathrm{HI}=\frac{PTV\_D2\%-PTV\_D98\%}{\mathrm{Dprescribed}}$ where Dprescribed = 60 Gy. The CN was calculated using the formula $\mathrm{CN}=\frac{{\left(TVri\right)}^{2}}{\left(TV*Vri\right)}$ where TV is the target volume, TV_ri_ is the target volume covered by the reference isodose (57 Gy) structure and V_ri_ the volume of the reference isodose structure [34]. The OARs were evaluated using the criteria listed in Supplementary Table 1. Additionally, volume parameters V10Gy, V20Gy, V30Gy, V40Gy and V50Gy were reported for lungs and heart, as well as dose to the substructures of the heart. These were ventricle left and right (V5Gy, V15Gy, V30Gy, Dmax), atrium left and right (Dmax), pulmonary artery (V40Gy, Dmax), superior vena cava (Dmean, Dmax), left anterior descending artery (LAD), right coronary artery (RCA), circumflex artery (V15Gy, Dmax), sinoatrial node and atrioventricular node (Dmean, Dmax) [20], [21], [32], [35].

### 2.4 NTCP analysis

To investigate the clinical relevance of the dose differences, NTCP values were calculated for the three most common toxicities in lung cancer, i.e, radiation pneumonitis, acute esophageal toxicity and two-year overall mortality. This study used the lung cancer NTCP models as described and recommended by the Dutch society of Radiotherapy and Oncology: for each toxicity the most reliable NTCP models were selected according to the TRIPOD criteria and recalibrated for the Dutch population [36]. The first NTCP model predicted the probability of grade ≥2 radiation pneumonitis in the first six months after treatment [23], [36]. NTCPs were calculated for a low and high risk scenario (see Supplementary Table 2 for the scenario settings). The second NTCP model predicted the probability of grade ≥2 acute esophageal toxicity [24], [36] and the third model predicted two-year overall mortality [25], [36]. Model formulas, parameters and their variables are depicted in Table 2. For each patient, the delta NTCP (ΔNTCP) was calculated for RP, AET and two-year overall mortality between the four treatment plans.

**Table 2:** Formulas of the three NTCP models and the parameter variables within the NTCP models.

**NTCP**$=\frac{1}{1+e^{-S}}$**with S:**	
Grade ≥2 Radiation Pneumonitis	S = -4.12 + 0.138 * MLD – 0.3711 * (Smoking: Stopped) − 0.478 * (Smoking: Active) + 0.8198 * (Pulmonary comorbidity) + 0.6259 * (Tumor location) + 0.5068 * (Age) + 0.47 * (Sequential chemotherapy)
Grade ≥2 Acute Esophageal Toxicity	S = -3.634 + 1.496 * ln(MED) − 0.0297 * (Interval start-stop RT)
Grade 5 Two-year overall mortality	S = -1.3409 + 0.0590 * SQRT(GTV) + 0.2635 * SQRT(MHD)

**Model parameters and variables**	

MLD	Mean lung dose (Gy) based on delineated total lungs minus GTV
Smoking: Stopped	Stopped smoking = 1, never or active smoker = 0
Smoking: Active	Active smoker = 1, never smoked or stopped = 0
Pulmonary comorbidity	COPD or other pre-existent lung disease = 1, None = 0
Tumor location	Middle or lower lobe = 1, upper lobe = 0
Age	≥63 years = 1, <63 years = 0
Sequential chemotherapy	Yes = 1, No = 0
MED	Mean esophageal dose (Gy)
Interval start-stop RT	Interval of radiotherapy treatment in days
GTV	Volume (cc) of total GTV (primary and nodes)
MHD	Mean heart dose (Gy)

### 2.5 Statistical analysis

To determine the optimal technique, ΔNTCP thresholds were equal to the patient selection criteria for proton therapy as used by the model-based approach in the Netherlands [37]. Patients were selected for proton therapy if the ΔNTCP between the photon and proton treatment plan exceeded a predetermined threshold. These thresholds depended on the severity of the toxicity and for this study meant a ΔNTCP threshold of 10 percent point (pp) for grade ≥2 toxicities RP and AET and 2 pp for grade 5 two-year overall mortality. Statistical significance was tested using a Wilcoxon signed-rank Test (IBM SPSS Statistics for Windows, version 26, IBM Corp., Armonk, NY, USA) and p-values < 0.05 were considered significant. This retrospective planning study was approved by the Internal Review Board and has a RATING score of 87% [38].

## 3 Results

**Fig. 1:**
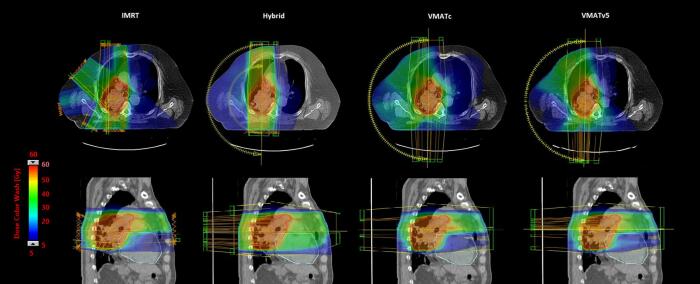
Example of the four treatment techniques used in this study. Transversal and sagittal views are shown to illustrate differences in doses to the lungs and heart.

**Fig. 2:**
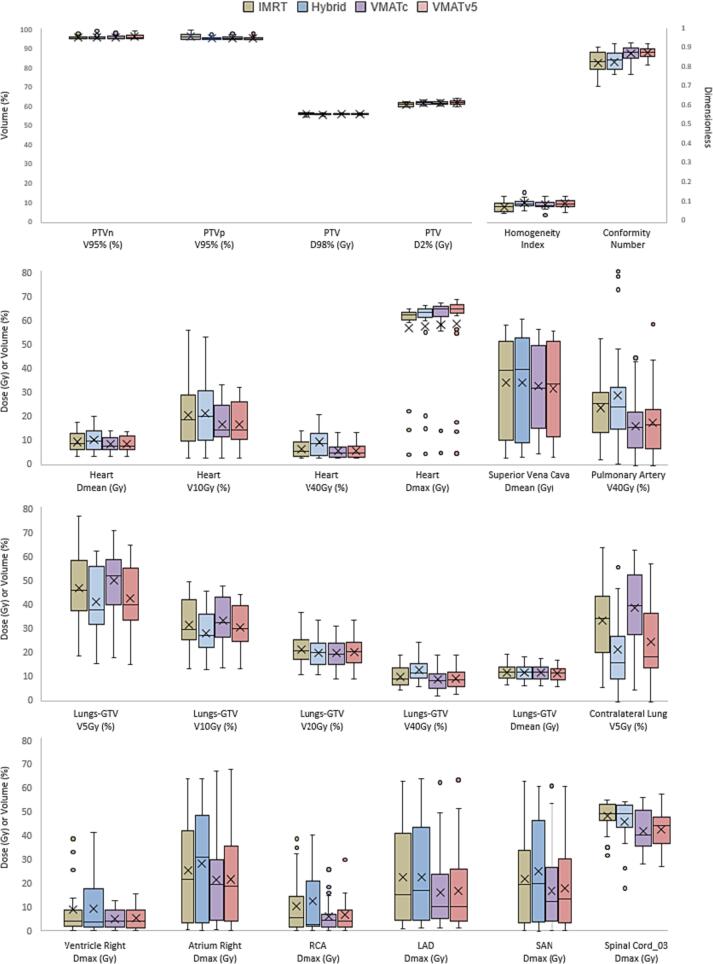
Boxplots of target volumes and a selection of organs at risk doses. Crosses are means, horizontal middle lines are medians. Circles are outliers and are defined as exceeding 1.5 times the interquartile range from the lower or upper quartile respectively. IMRT: Intensity Modulated Radiation Therapy, VMAT: Volumetric Modulated Arc Therapy, PTV: Planning Target Volume, RCA: Right Coronary Artery, LAD: Left Anterior Descending Artery, SAN: Sinoatrial Node.

An example of the four techniques is provided in Fig. 1. All DVH parameters for the four techniques are listed in Supplementary Table 3 and Supplementary Fig. 2, and a selection of the results is shown in Fig. 2.

### 3.1 Target coverage

The target coverage parameters of the PTV showed that hybrid plans scored worst for the D98% with a median of 55.8 Gy. The D2% was lowest in IMRT plans with a median of 61.5 Gy. VMAT plans were most conformal with a CN of 0.9. Absolute differences between the techniques were small (<1.1 Gy and <1%) and the coverage of the PTV volumes was sufficient (at least 95%) in all four techniques.

### 3.2 Organs at risk

The two VMAT techniques showed the lowest MHD, 4.9 Gy for VMAT_C_ and 5.0 Gy for VMAT_V5_. IMRT and hybrid plans had a significant higher MHD with hybrid plans having the highest MHD of 7.2 Gy. All the other volume parameters, except for the V5Gy were also lowest in the VMAT plans. IMRT scored better when compared to the hybrid technique. The maximum dose in the heart was the lowest in the IMRT plans.

The MLD was significantly lowest in VMAT_V5_ (19.9 Gy) and highest in the hybrid plans (12.5 Gy). The V20Gy was very similar between the four techniques and ranged from 20% (VMAT_C_) to 21% (IMRT). The low dose parameters V5Gy and V10Gy of the total lungs were significantly lowest in the hybrid plans compared to VMATc and IMRT. Hybrid and VMAT_V5_ plans had a median V5Gy of the total lungs of 38% and 40% respectively. VMAT_C_ plans performed worst with a median V5Gy of 52%. The lower VXGy parameters (V5Gy, V10Gy) were lowest in the hybrid technique, but this advantage disappeared for the higher VXGy parameters (V30Gy, V40Gy, V50Gy) where the VMAT plans scored better. The V5Gy of the contralateral lung was lowest in the hybrid (16%) and VMAT_V5_ (18%) plans. VMAT_C_ plans had the highest contralateral dose (39%), with a maximum difference of 17% in favor of the hybrid technique.

The mean esophageal dose was highest in IMRT with a median of 18.8 Gy compared to 17.2 Gy for VMATc. Absolute mean differences between the other techniques were small, at most 0.9 Gy. The maximum dose in the esophagus was similar between the techniques. The lowest maximum dose in the spinal cord and mediastinal envelope was reached in the VMAT- and IMRT plans respectively.

### 3.3 Substructures of the heart

The dose to the substructures of the heart depended a lot on the location of the tumor. Most differences were not significant and small (at most 1.8 Gy). Largest and significant differences in substructure dose between the techniques were observed in the right atrium, superior vena cava, pulmonary artery, LAD and sinoatrial node, mostly in favor of the VMAT_C/V5_ techniques.

### 3.4 Normal tissue complication probabilities

NTCP values were calculated for each technique and are shown in Table 3. In general, patients had a median probability to develop grade ≥2 RP with no risk factors between 3.6 and 3.7%, with all risk factors between 37.3 and 39.3%, grade ≥2 AET between 34.7 and 37.8% and grade ≥5 mortality of 46.3–48.9% all in favor of the two VMAT techniques. Although some of the differences in NTCP were significant, the median differences between the four techniques did not exceed the 2 pp or 10 pp thresholds for grade ≥5 and grade ≥2 toxicity, respectively.

**Table 3:** Median normal tissue complication probabilities for radiation pneumonitis, acute esophageal toxicity and two-year overall mortality for the IMRT, hybrid, VMATc and VMATv5 treatment plans and median differences in NTCP between the techniques. Red numbers indicate that the P-value was <0.05. Bold numbers indicate that the technique had the lowest mean NTCP value. See Supplementary Table 2 for the two scenario settings. IMRT: Intensity Modulated Radiotherapy, VMAT: Volumetric Modulated Arc Therapy, RP: Radiation Pneumonitis, AET: Acute Esophageal Toxicity.

**Fig. 3:**
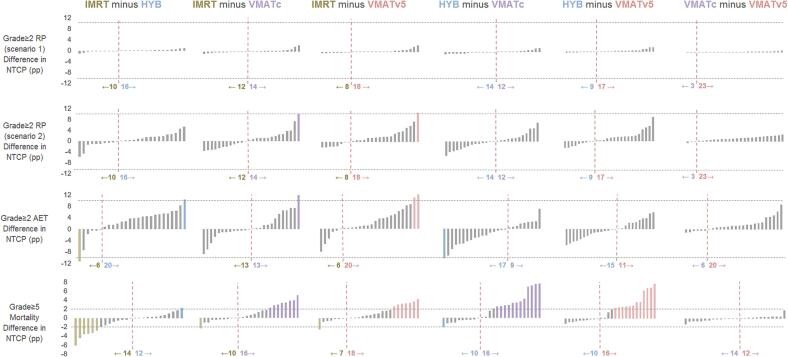
Distribution of individual patient differences for each treatment technique and toxicity. Each bar represents a patient. The height of the bar indicates the difference in NTCP (ΔNTCP) between the techniques. The colored bars indicate the treatment techniques that performed more than 10 pp or 2 pp better for grade ≥2 or grade 5 toxicity respectively. The horizontal dashed grey lines represent the threshold. Grey bars indicate that the delta did not exceed the threshold. The vertical dashed line shows the tipping point, where one technique is better than the other technique. IMRT: Intensity Modulated Radiation Therapy, VMAT: Volumetric Modulated Arc Therapy, NTCP: Normal Tissue Complication Probability, RP: Radiation Pneumonitis, AET: Acute Esophageal Toxicity, pp: percent point. A summarized overview is given in Supplementary Table 4.

Individual patient differences for all plan comparisons are shown in Fig. 3. A summarized overview is given in Supplementary Table 4. For grade 5 mortality the ΔNTCP exceeded the 2 pp threshold in 51/156 (33%) of the evaluated plan comparisons. In 41/51 of these plan comparisons this was in favor of VMAT, compared to 8/51 in favor of IMRT and 2/51 in favor of the hybrid technique. For RP scenario 1, the ΔNTCP did not exceed the 10 pp threshold. RP scenario 2 showed higher ΔNTCP values, and the threshold of 10 pp was reached in 2/156 (1%) of the plan comparisons in favor of VMAT. For grade ≥2 AET the ΔNTCP exceeded the 10 pp threshold in 6/156 (4%) of all plan comparisons and showed a slight advantage for the hybrid and VMAT_V5_ technique.

## 4 Discussion

Overall, the results of this study indicate that the median dose differences between the four techniques had no large effect on the probability to develop the three most common radiation induced side effects. However, the evaluation of individual patient differences showed that the dose differences in the plan comparisons were large enough to cause a ΔNTCP that exceeded the threshold of 2 pp or 10 pp for grade 5 (mortality) and grade ≥2 toxicity (RP and AET), respectively, in a substantial percentage of plan comparisons (38%).

The largest dose differences were observed in the low dose parameters of the lungs (V5Gy for the total lungs and contralateral lung) in favor of the hybrid and VMAT_V5_ technique. However, current NTCP models for lung cancer related toxicities do not include the V5Gy for the total lungs or the contralateral lung. Clinically, the V5Gy of both lungs is preferred to be below 60%. All four techniques had a median V5Gy <60%. In 11/104 treatment plans the V5Gy of the total lungs was higher than 60% in four VMAT_C_, one hybrid, four IMRT and one VMAT_V5_ treatment plan.

Combining the results of the DVH and NTCP analysis, the VMAT_V5_ technique outperformed the other techniques for most patients (See Supplementary Table 3 and Fig. 3). The V5Gy of the lungs and contralateral lung were similar to the hybrid technique (median difference of 1.8% and 3.3%, respectively, in favor of the hybrid technique), and for mortality the NTCP was similar to VMAT_C_ (median difference of 0.1 pp in favor of the VMAT_C_ technique), combining the best aspects of the VMAT_C_ and hybrid technique. This is different from other studies where VMAT performed worst for lung V5Gy and V10Gy. Combining a strict constraint for the heart (<10 Gy) and stricter lung objectives show that it is possible to achieve both low lung and heart dose simultaneously using VMAT. IMRT and hybrid plans were not further optimized since the heart dose was already higher compared to VMAT and pushing for lower lung dose would likely increase heart dose even more, but this could be investigated further.

The DVH differences between the IMRT, hybrid and VMAT_C_ techniques were similar to the results of previous planning studies, reporting lower lung and contralateral lung doses (V5Gy, V10Gy) in IMRT/hybrid plans and lower heart doses in VMAT plans [7], [10], [11], [14]. The recent study of Li et al. [39] also compared four photon treatment techniques and reported similar results favoring IMRT and VMAT for better dose coverage and lower organs at risk dose compared to 3DCRT and a hybrid IMRT technique. Overall, the V5Gy in their study was lower compared to the V5Gy in our study and their MHD was higher, which might be caused by different optimization priorities and their higher dose constraint for the heart (<26 Gy versus <10 Gy).

The low lung doses observed in the hybrid technique are in agreement with the results of the study of Verbakel et al. [3]. They found that the hybrid technique was preferable to a full IMRT or VMAT technique to spare the dose to the lungs. Their mean V20Gy and contralateral lung V5Gy values were different compared to our study, which could be caused by their patient population (a median PTV of 779 cc versus 416 cc in our study). Their study did not report the dose to the heart, which in our study was the highest in the hybrid technique. Similar differences in lung and heart dose were reported in the study of Zhang et al. [10] where IMRT was recommended for patients with poor pulmonary function and VMAT was recommended for patients with poor cardiac conditions. The dose to the heart in their study was much higher compared to our study. This could be caused by delineation differences or different priorities in the optimization process.

Dose to the heart is receiving more attention given increasing evidence for a correlation between cardiac dose and cardiac toxicity and survival [20], [40]. However, this has not yet lead to updated clinical guidelines. Our current treatment optimization strategy aims for a MHD <10 Gy which resulted in a median MHD between 4.9 Gy (VMAT_C_) and 7.2 Gy (hybrid). DVH parameters of the heart substructures that were significantly associated with cardiac events or overall survival in recently published studies [20], [21] were compared to the doses in this study. The median doses to the left ventricle (V15Gy), right atrium (Dmax, only for VMAT), pulmonary artery (V40Gy), RCA (V15Gy), LAD (V15Gy), and circumflex artery in our plans were below their criteria. However, since dose to the substructures is tumor location dependent there is a group of patients receiving doses larger than these criteria. This indicates that our treatment plans performed well, but potentially could be further optimized for some substructures for individual patients. More planning studies and better-defined criteria are needed to be able to use these constraints during treatment planning optimization.

NTCP models provide insight in radiation induced toxicities and are employed in this study to put intra-patient dose differences into clinical perspective. The NTCP thresholds in this study were based on photon-proton patient stratification guidelines in the Netherlands, that consider the severity of the toxicity (>10 pp for grade ≥2, >2 pp for grade = 5), as a conservative approach [37]. Evidence of the performance of these models is still limited and further validation is important. Improved models can distinguish between clinical relevance and irrelevance better, and lead to improved treatment decisions for individual patients to reduce toxicities, e.g., selecting the best treatment planning technique.

In conclusion, the DVH analysis in this study showed a trade-off between the V5Gy of the lungs (hybrid/VMAT_V5_) and the MHD (VMAT_C/V5_). The NTCP analysis showed that VMAT is favored, predominantly due to a decreased probability of mortality. VMAT_V5_ was ultimately preferred combining low lung and heart doses, as well as low NTCPs, and has therefore been implemented in our clinic.

## CRediT authorship contribution statement

**Djoya Hattu:** Conceptualization, Methodology, Validation, Formal analysis, Investigation, Resources, Data curation, Writing – original draft, Visualization, Project administration. **Daisy Emans:** Investigation. **Judith van der Stoep:** Investigation. **Richard Canters:** Software, Writing – review & editing. **Judith van Loon:** Conceptualization, Writing – review & editing, Supervision. **Dirk De Ruysscher:** Conceptualization, Writing – review & editing, Supervision.

## Declaration of Competing Interest

The authors declare that they have no known competing financial interests or personal relationships that could have appeared to influence the work reported in this paper.
