# Supplementary material for: Comparison of photon intensity modulated, hybrid and volumetric modulated arc radiation treatment techniques in locally advanced non-small cell lung cancer
Source: Phys Imaging Radiat Oncol. 2023 Nov 18;28:100519. doi: 10.1016/j.phro.2023.100519 (PMC10726236; doi:10.1016/j.phro.2023.100519)
Supplement: Supplementary data 1 [file mmc1.pdf]

Supplementary Materials

Supplementary Figure 1: An example of the DVH with markers showing the objective functions for the total lungs, contralateral lung and planning target volume in the optimization. The left plot shows the VMATc DVH and objectives and the right plot the VMATv5 DVH/objectives with slightly stricter objectives for the lungs. Optimization objectives in the VMATc plot (left) are similar to the optimization objectives used in the IMRT and hybrid plans. Arrows pointing north-east are lower objectives, arrows pointing south-west are upper objectives, diamonds on the x-axis are mean objective values. Dark blue: contralateral lung, Light Purple: Lungs-PTV, Red: PTV. Objectives for the heart and esophagus were similar for all four treatment techniques. In this example the total lungs V5Gy was reduced with 7%, the contralateral lung V5Gy was reduced with 12.6% and the mean heart dose increased with 0.4Gy in the VMATv5 plan compared to VMATc.

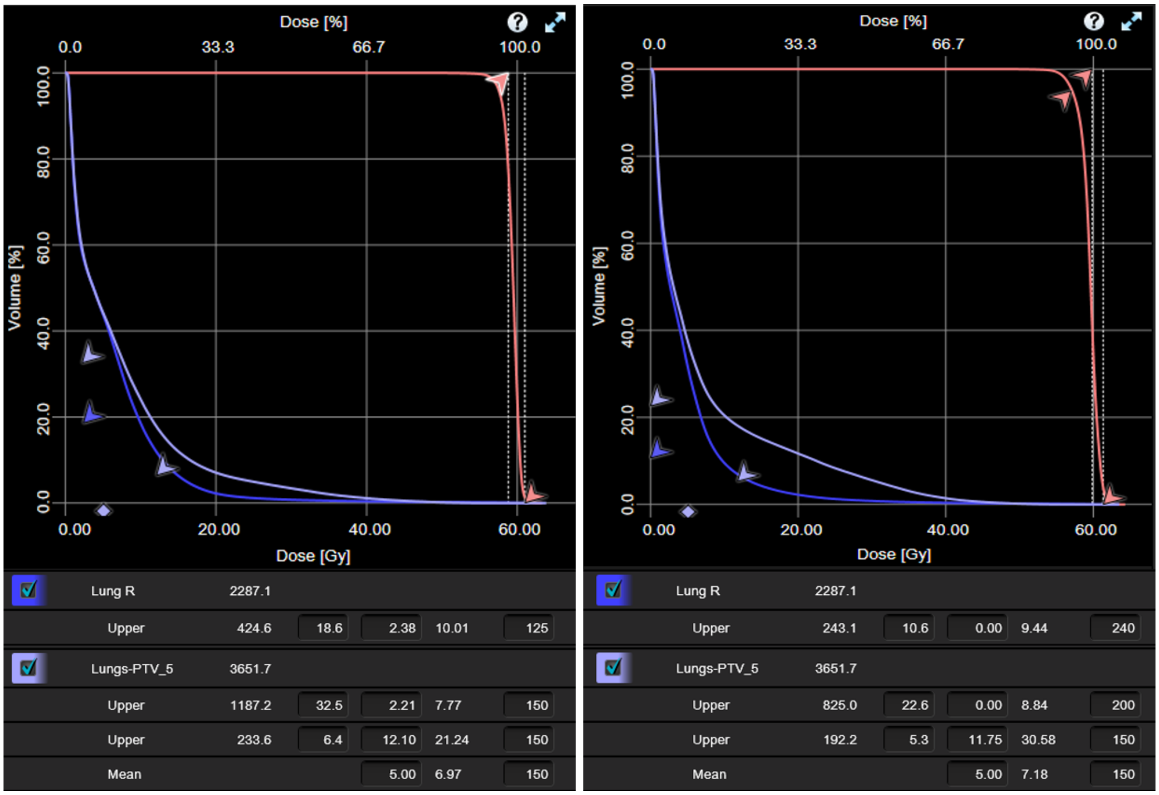

Supplementary Figure 2a: Boxplots of all target volumes and organs at risk doses. Crosses are means, horizontal middle lines are medians. Circles are outliers and are defined as exceeding 1.5 times the interquartile range from the lower or upper quartile respectively. IMRT: Intensity Modulated Radiation Therapy, VMAT: Volumetric Modulated Arc Therapy, GTV: Gross Tumor Volume, MedEnv_05: Mediastinal envelope with a 5mm expansion, PTV: Planning Target Volume.

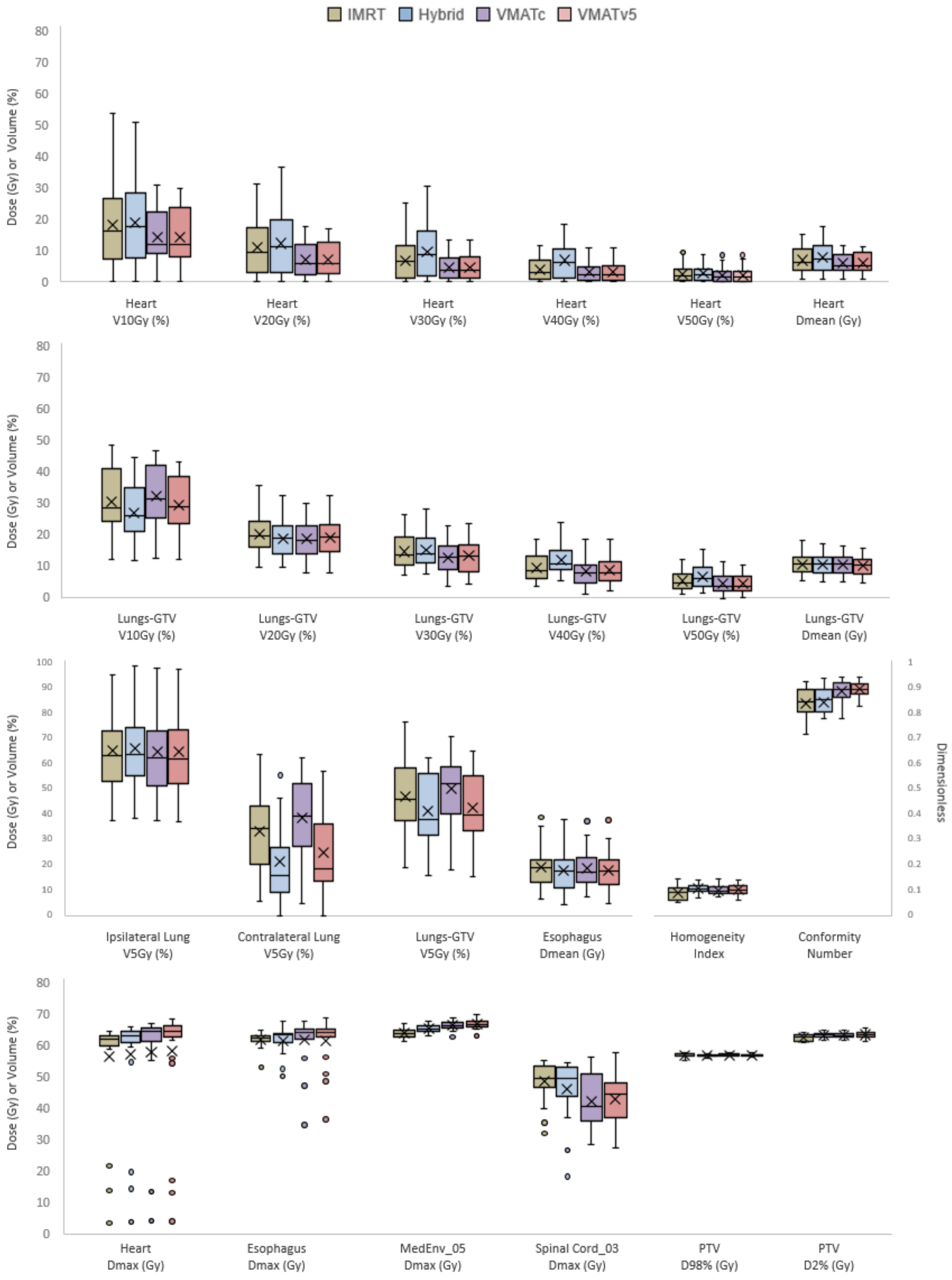

Supplementary Figure 2b: Boxplots of all substructures of the heart. Crosses are means, horizontal middle lines are medians. Circles are outliers and are defined as exceeding 1.5 times the interquartile range from the lower or upper quartile respectively. IMRT: Intensity Modulated Radiation Therapy, VMAT: Volumetric Modulated Arc Therapy. RCA: Right Coronary Artery, LAD: Left Anterior Descending artery, CFLX: circumflex artery, SAN: Sinoatrial node, AVN: Atrioventricular node.

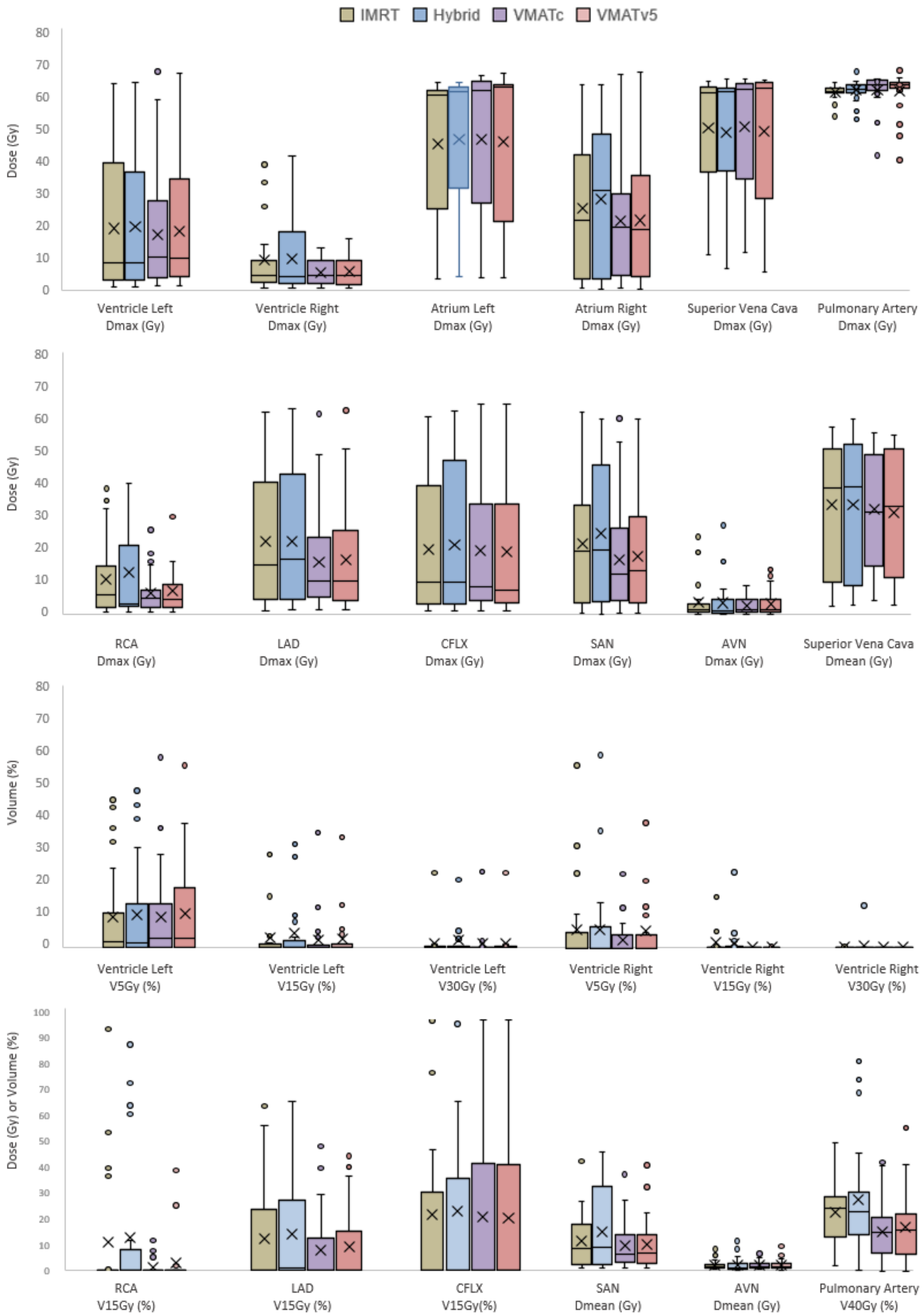

Supplementary Table 1: Clinical goals used for plan evaluation and the planning protocol. PTVnsum: Planning Target Volume of the nodes, PTVp: Planning Target Volume of the primary tumor, GTV: Gross Tumor Volume, Mediastinum_05: planning risk volume defined as the mediastinum expanded with 5mm, Spinal_Cord_03: planning risk volume defined as the spinal cord expanded with 3mm.

**OAR**	**Evaluation criteria**		**Type of optimization objectives**
PTVnsum	V95%	≥ 95%	Lower
	Dmax	≤ 115%	Upper
PTVp	V95%	≥ 95%	Lower
	Dmax	≤ 115%	Upper
Body	Dmax	≤ 115%	Upper
Heart	Dmax	< 69Gy	Upper
Heart	Dmean	< 10Gy	Mean
Lungs – GTV	Dmean	< 20Gy	Upper, mean
Lungs – GTV	V5Gy	< 60%	Upper
Mediastinum_05	Dmax	< 69Gy	Upper
Spinal Cord_03	Dmax	< 54Gy	Upper

**Planning protocol**	
1	Fractionation 30x2Gy
2	TrueBeam STx, Acuros v15.5.11
3	Beam setup: Follow the study protocol for IMRT, hybrid and VMAT planning technique.<br>IMRT: start with the standard setup of 5 beams (Left: 0, 50, 100, 150, 179 or Right: 181, 210, 260, 310, 0) and adjust the beam directions if necessary. In this study the standard beam angles were not adjusted for any patient.<br>HYBRID: start with the AP/PA beams that account for 2/3 of the dose and add a half arc on the affected side, add a second half arc if this is necessary. The PTV sizes in this study required a secondary arc in 23/26 of the patients.<br>VMAT: start with two half arcs (0-181 right sided or 0-179 left sided) on the affected side. For all arcs it was allowed to extend the starting/end point over the midline if this was necessary. This was found necessary in 2/26 patients.
4	Optimization: Order of OARs in the optimization: heart (MHD), lungs (MLD, V5Gy), esophagus. Multiple optimization rounds were allowed until the treatment planner was satisfied and the above mentioned evaluation criteria were met. For VMATv5 there was an extra focus on further lowering the V5Gy after the previous process was performed. Treatment planners were made aware that further lowering the V5Gy could cause an increase in MHD and more spread out dose (V80%) but that this was allowed to get to a balance between lung and heart dose.
5	Normalization: For both PTVn as PTVp the V95% should be at least 95%. Since it is not possible to normalize on two structures separately, the PTV with the lowest coverage was normalized to a V95% of 95%. Because of this the other PTV will automatically have a V95% larger than 95%.
6	If constraints were not achievable this was discussed with a physician.
7	Treatment plans were approved by a physician.

Supplementary Table 2: Overview of the variables in the radiation pneumonitis grade ≥2 NTCP model and the different input values of each scenario.

	**Scenario 1 (no risk factors)**	**Scenario 2 (all risk factors)**
Smoking: stopped	Stopped =1	Never or active = 0
Smoking: active	Active smoker = 1	Never or stopped = 0
Pulmonairy comorbidity	None = 0	COPD or other pre-existent lung disease = 1
Tumor location	Upper lobe = 0	Middle-/Lower lobe = 1
Age	<63 years = 0	≥63 years = 1
Sequential chemotherapy	No = 0	Yes = 1

Supplementary Table 3: Planning target volume and organs at risk dose parameters for the IMRT, hybrid, VMATc and the VMATv5 plans. For all dose parameters, the median and interquartile range of each technique are given. Also, absolute median differences between the techniques are shown. Red numbers indicate significant differences (p<0.05). Be aware that substructures of the heart are very small and therefore very location dependent so the median dose might not always be representative for all patients. PTV: Planning Target Volume, CN: Conformity Number, HI: Homogeneity Index, GTV: Gross Target Volume, MedEnv_05: Mediastinal Envelope with a 5mm expansion, SVC: Superior Vena Cava, RCA: Right Coronary Artery, LAD: Left Anterior Descending coronary artery, CFLX: Circumflex coronary artery, SAN: Sino Atrial Node, AVN: Atrioventricular node.

	Treatment technique				Median differences between the techniques					
	IMRT	HYBRID	VMATc	VMATv5	IMRT – HYB	IMRT – VMATc	IMRT – VMATv5	HYB – VMATc	HYB – VMATv5	VMATc – VMATv5
PTV D98% (Gy)	56.3 (55.6 - 56.5)	55.8 (55.6 - 56.0)	56.0 (55.9 -56.2)	56.0 (55.8 - 56.1)	0.3	0.0	0.2	-0.2	-0.2	0.0
PTV D2% (Gy)	61.5 (60.3-62.3)	62.3 (61.7 - 62.7)	62.0 (61.6 - 62.7)	62.2 61.6 - 63.1)	-1.1	-0.8	-1.0	0.2	0.0	-0.4
PTVn V95% (%)	95.0 (95.0 - 95.8)	95.0 (95.0 - 95.5)	95.0 (95.0 - 95.9)	95.0 (95.0 - 96.5)	0.0	0.0	0.0	0.0	0.0	0.0
PTVp V95% (%)	96.1 (95.0 - 97.5)	95.3 (95.0 - 95.8)	95.1 (95.0 - 95.9)	95.0 (95.0 - 95.6)	0.0	0.0	0.4	0.0	0.1	0.0
CN	0.8 (0.8 - 0.9)	0.8 (0.8 - 0.9)	0.9 (0.9 - 0.9)	0.9 (0.9 - 0.9)	0.0	0.0	0.0	0.0	0.0	0.0
HI	0.1 (0.1 - 0.1)	0.1 (0.1 - 0.1)	0.1 (0.1 - 0.1)	0.1 (0.1 - 0.1)	0.0	0.0	0.0	0.0	0.0	0.0
Heart Dmean (Gy)	5.9 (3.9 - 10.1)	7.2 (4.0 - 11.1)	4.9 (4.0 - 8.5)	5.0 (4.0 - 9.0)	-0.1	0.3	0.4	1.3	1.3	-0.1
Heart V5Gy (%)	23.1 (15.2 - 40.0)	25.6 (14.4 - 40.8)	27.0 (16.0 - 38.8)	25.6 (15.0 - 38.6)	-1.0	-1.0	-1.0	-0.5	-0.4	0.1
Heart V10Gy (%)	15.8 (7.9 - 25.3)	17.4 (8.0 - 27.5)	11.5 (9.0 - 21.1)	11.7 (8.3 - 22.9)	-0.2	0.6	1.5	2.6	1.8	0.0
Heart V20Gy (%)	9.4 (3.5 - 16.6)	10.8 (3.5 - 19.2)	5.7 (2.7 - 10.8)	5.7 (2..7 - 11.9)	-0.2	2.1	2.1	4.0	3.4	0.0
Heart V30Gy (%)	6.4 (1.5 - 10.5)	8.4 (2.4 - 15.0)	3.6 (1.2 - 7.2)	3.8 (1.3 - 7.6)	-0.7	0.8	0.8	4.2	3.6	-0.1
Heart V40Gy (%)	2.9 (0.7 - 6.1)	6.1 (1.7 - 10.2)	2.2 (0.5 - 4.5)	2.1 (0.6 - 4.9)	-1.9	0.5	0.3	3.7	3.3	0.0
Heart V50Gy (%)	1.7 (0.3 - 3.5)	2.4 (0.5 - 4.0)	1.3 (0.2 - 3.0)	1.4 (0.2 - 3.0)	-0.2	0.1	0.1	0.5	0.5	0.0
Heart Dmax (Gy)	61.4 (59.5 - 62.4)	62.3 (60.4 - 63.6)	63.6 (61.4 - 64.7)	63.7 (62.2 - 65.3)	-0.7	-2.2	-2.3	-0.7	-1.1	-0.4
Lungs-GTV Dmean (Gy)	12.3 (10.0 - 14.3)	12.5 (9.9 - 14.1)	12..2 (9.9 - 14.3)	11.9 (9.5 - 13.7)	0.1	0.1	0.4	0.0	0.4	0.4
Lungs-GTV V5Gy (%)	45.6 (37-6 - 57.6)	37.7 (32.3 - 55.2)	51.5 (40.5 - 57.7)	39.6 (34.0 - 53.7)	5.1	-3.9	3.3	-9.0	-1.8	6.3
Lungs-GTV V10Gy (%)	29.5 (26.1 -41.1)	27.1 (23.0 - 34.5)	32.5 (26.6 - 41.8)	30.1 (25.3 - 38.8)	3.1	-1.2	0.9	-4.5	-2.4	2.2
Lungs-GTV V20Gy (%)	21.1 (18.0 - 24.9)	20.2 (15.6 - 23.8)	19.6 (16.1 - 24.0)	20.5 (16.3 - 24.2)	1.4	0.9	0.5	-0.4	-0.4	-0.3
Lungs-GTV V30Gy (%)	15.0 (12.2 - 20.4)	15.5 (13.0 - 20.0)	14.3 (10.9 - 17.5)	14.8 (10.4 - 17.8)	-0.2	1.9	1.0	2.4	1.2	-0.7
Lungs-GTV V40Gy (%)	9.8 (7.8 - 14.2)	12.2 (10.2 - 16.1)	9.3 (6.3 - 11.4)	9.4 (7.0 - 12.7)	-2.4	1.2	1.0	4.0	3.1	-0.3
Lungs-GTV V50Gy (%)	6.2 (4.6 - 8.7)	7.6 (5.3 - 10.4)	5.1 (4.1 - 7.9)	5.2 (3.9 - 7.9)	-1.1	0.7	0.7	1.4	1.6	0.1
Contralateral V5Gy (%)	34.1 (20.7 - 41.2)	16.0 (9.8 - 25.1)	39.2 (28.0 - 50.5)	18.4 (14.5 - 33.7)	11.5	-6.5	6.1	-17.3	-3.3	12.3
Esophagus Dmean (Gy)	18.8 (13.7 - 21.8)	17.5 (11.7 - 21.6)	17.2 (13.4 - 22.5)	17.4 (12.5 - 21.6)	1.9	0.0	0.9	-0.7	-0.2	0.6
Esophagus Dmax (Gy)	61.2 (60.2 - 61.9)	62.2 (60.3 - 62.6)	63.0 (61.3 - 63.9)	62.9 (61.9 - 64.1)	-0.5	-1.9	-1.7	-0.8	-0.6	-0.4
MedEnv_05 Dmax(Gy)	63.1 (62.1 - 63.9)	64.5 (64.0 - 65.3)	65.3 (65.0 - 66.4)	65.8 (65.0 - 66.6)	-1.4	-2.6	-2.9	-0.6	-0.8	-0.3
Spinal Cord_03 Dmax (Gy)	48.7 (46.3 - 52.5)	48.9 (43.7 - 51.6)	40.1 (35.7 - 49.7)	43.7 (36.9 – 47.1)	1.1	6.6	5.6	2.6	4.9	-0.6

	Treatment technique				Median differences between the techniques					
	IMRT	HYBRID	VMATc	VMATv5	IMRT - HYB	IMRT - VMATc	IMRT - VMATv5	HYB - VMATc	HYB - VMATv5	VMATc - VMATv5
Left Ventricle V5Gy (%)	1.7 (0.0 - 8.2)	1.1 (0.0 - 11.2)	2.6 (0.0 - 11.7)	2.5 (0.0 - 15.6)	-0.2	0.0	0.0	0.0	0.0	0.0
Left Ventricle V15Gy (%)	0.0 (0.0 - 0.8)	0.0 (0.0 - 1.6)	0.0 (0.0 - 0.3)	0.0 (0.0 - 0.5)	0.0	0.0	0.0	0.0	0.0	0.0
Left Ventricle V30Gy (%)	0.0 (0.0 - 0.1)	0.0 (0.0 - 0.0)	0.0 (0.0 - 0.0)	0.0 (0.0 - 0.0)	0.0	0.0	0.0	0.0	0.0	0.0
Left Ventricle Dmax (Gy)	8.6 (3.8 - 34.6)	8.5 (3.7 - 31.5)	10.2 (3.9 - 22.2)	10.0 (4.5 - 28.4)	-0.3	0.3	-0.1	0.3	-0.1	-0.1
Right Ventricle V5Gy (%)	0.0 (0.0 - 3.9)	0.0 (0.0 - 6.1)	0.0 (0.0 - 3.5)	0.0 (0.0 - 3.2)	0.0	0.0	0.0	0.0	0.0	0.0
Right Ventricle V15Gy (%)	0.0 (0.0 - 0.0)	0.0 (0.0 - 0.0)	0.0 (0.0 - 0.0)	0.0 (0.0 -0.0)	0.0	0.0	0.0	0.0	0.0	0.0
Right Ventricle V30Gy (%)	0.0 (0.0 - 0.0)	0.0 (0.0 - 0.0)	0.0 (0.0 - 0.0)	0.0 (0.0 - 0.0)	0.0	0.0	0.0	0.0	0.0	0.0
Right Ventricle Dmax (Gy)	4.6 (2.7 – 9.0)	4.2 (2.2 - 17.4)	4.5 (2.3 - 9.1)	4.5 (2.0 – 9.0)	0.1	0.3	0.6	0.4	0.4	-0.0
Left Atrium Dmax (Gy)	59.3 (26.7 - 60.8)	60.4 (38.1 - 61.7)	60.6 (27.2 - 63.4)	61.8 (23.4 - 62.4)	-0.8	-0.9	-1.5	-0.8	-1.2	0.0
Right Atrium Dmax (Gy)	21.6 (4.5 - 40.2)	30.6 (4..4 - 46.9)	19.4 (5.4 - 28.1)	19.0 (4.7 - 32.4)	-0.4	0.1	0.6	2.0	1.9	0.2
SVC Dmean (Gy)	38.8 (13.0 - 49.5)	38.8 (11.4 - 51.0)	31.2 (15.7 - 47.3)	33.1 (12.2 – 49.0)	0.5	1.8	2.1	1.7	2.5	0.5
SVC Dmax (Gy)	60.0 (40.4 - 61.5)	60.4 (38.8 - 61.5)	61.0 (35.3 - 62.8)	61.3 (29.9 – 3.0)	0.5	-0.7	-0.1	-1.1	-0.9	0.3
Pulmonary Artery V40Gy (%)	25.1 (14.2 – 29.4)	23.7 (16.3 – 29.6)	15.4 (7.6 - 20.6)	16.4 (7.3 - 21.5)	0.4	7.2	7.1	8.1	6.0	-0.1
Pulmonary Artery Dmax (Gy)	60.5 (60.1 - 61.4)	61.0 (60.3 - 62.2)	62.6 (61.1 - 63.6)	62.3 (61.5 - 63.2)	-0.5	-1.9	-1.4	-1.0	-0.6	0.4
RCA V15Gy (%)	0.0 (0.0 - 0.0)	0.0 (0.0 – 8.0)	0.0 (0.0 - 0.0)	0.0 (0.0 -0.0)	0.0	0.0	0.0	0.0	0.0	0.0
RCA Dmax (Gy)	6.0 (2.3 - 14.1)	3.4 (2.8 - 17.9)	5.2 (2.4 - 7.0)	4.6 (2.4 - 8.5)	-0.8	0.6	1.1	0.6	0.4	-0.1
LAD V15Gy (%)	0.0 (0.0 - 21.1)	0.6 (0.0 - 25.5)	0.0 (0.0 - 7.2)	0.0 (0.0 - 10.2)	0.0	0.0	0.0	0.0	0.0	0.0
LAD Dmax (Gy)	15.6 (5.4 - 39.9)	17.1 (5.2 - 41.3)	10.5 (5.9 - 22.9)	10.7 (5.2 - 25.3)	-1.0	3.5	2.9	2.4	2.1	-0.7
CFLX V15Gy (%)	0.0 (0.0 - 23.2)	0.0 (0.0 - 21.5)	0.0 (0.0 - 32.6)	0.0 (0.0 - 33.0)	0.0	0.0	0.0	0.0	0.0	0.0
CFLX Dmax (Gy)	10.2 (4.5 - 37.2)	10.2 (4.3 - 41.2)	8.9 (5.0 - 27.1)	7.8 (4.4 - 27.6)	-1.5	-0.9	0.2	-0.2	-0.0	0.1
SAN Dmean (Gy)	8.7 (2.6 - 17.0)	8.9 (2.6 - 30.3)	6.3 (3.1 - 13.4)	6.8 (2.8 - 12.8)	-0.5	0.7	0.7	1.4	1.1	-0.1
SAN Dmax (Gy)	19.7 (5.7 - 32.3)	19.9 (5.8 - 44.2)	12.6 (5.2 - 25.5)	13.9 (4.3 - 27.3)	-0.8	4.2	2.9	2.8	1.5	-0.2
AVN Dmean (Gy)	1.6 (1.0 - 2.4)	1.4 (0.9 - 2.7)	1.5 (1.0 - 2.8)	1.4 (1.0 - 2.5)	0.2	-0.1	0.0	-0.2	-0.2	0.0
AVN Dmax (Gy)	2.0 (1.3 - 4.5)	1.8 ((1.1 - 4.5)	1.9 (1.2 - 4.8)	1.9 (1.2 - 4.5)	0.2	-0.1	0.0	-0.3	-0.2	0.0

Supplementary Table 4: Number of patients with a ΔNTCP difference exceeding the thresholds of ±2pp and ±10pp for grade 2 and grade 5 toxicities respectively. The colors of the boxes indicate which technique performed better (IMRT, hybrid, VMATc, VMATv5) the number indicates for how many patients. Grey boxes indicate that the difference in NTCP was less than the predefined thresholds.

		IMRT - HYB	IMRT - VMATc	IMRT - VMATv5	HYB - VMATc	HYB - VMATv5	VMATc - VMATv5	Total
**Grade ≥2 Radiation Pneumonitis Scenario 1**								
# Patients	ΔNTCP < -10%	0	0	0	0	0	0	0
# Patients	ΔNTCP > 10%	0	0	0	0	0	0	0
# Patients	-10% < ΔNTCP < 10%	26	26	26	26	26	26	156
**Grade ≥2 Radiation Pneumonitis Scenario 2**								
# Patients	ΔNTCP < -10%	0	0	0	0	0	0	0
# Patients	ΔNTCP > 10%	0	1	1	0	0	0	2
# Patients	-10% < ΔNTCP < 10%	26	25	25	26	26	26	154
**Grade ≥2 Acute Esophageal Toxicity**								
# Patients	ΔNTCP < -10%	1	0	0	1	0	0	2
# Patients	ΔNTCP > 10%	1	1	2	0	0	0	4
# Patients	-10% < ΔNTCP < 10%	24	25	24	25	26	26	150
**Grade 5 Two-year overall mortality**								
# Patients	ΔNTCP < -2%	6	1	1	1	0	0	9
# Patients	ΔNTCP > 2%	1	8	7	13	12	1	42
# Patients	-2% < ΔNTCP < 2%	19	17	18	12	14	25	105
